# A Self‐Amplifying ROS‐Responsive Nanoplatform for Simultaneous Cuproptosis and Cancer Immunotherapy

**DOI:** 10.1002/advs.202401047

**Published:** 2024-04-03

**Authors:** Hangyi Wu, Zhenhai Zhang, Yanni Cao, Yuhan Hu, Yi Li, Lanyi Zhang, Xinyi Cao, Haitong Wen, Youwen Zhang, Huixia Lv, Xin Jin

**Affiliations:** ^1^ Department of Pharmaceutics China Pharmaceutical University Nanjing 211198 China; ^2^ Jiangsu Province Academy of Traditional Chinese Medicine Nanjing 210023 China; ^3^ Department of Pharmaceutics The Affiliated Suqian First People's Hospital of Nanjing Medical University Suqian Jiangsu 223800 China

**Keywords:** cinnamaldehyde, cuproptosis, elesclomol, immunogenic cell death, ROS‐responsive, self‐amplifying nanoplatform

## Abstract

Cuproptosis is an emerging cell death pathway that depends on the intracellular Cu ions. Elesclomol (ES) as an efficient Cu ionophore can specifically transport Cu into mitochondria and trigger cuproptosis. However, ES can be rapidly removed and metabolized during intravenous administration, leading to a short half‐life and limited tumor accumulation, which hampers its clinical application. Here, the study develops a reactive oxygen species (ROS)‐responsive polymer (PCP) based on cinnamaldehyde (CA) and polyethylene glycol (PEG) to encapsulate ES‐Cu compound (EC), forming ECPCP. ECPCP significantly prolongs the systemic circulation of EC and enhances its tumor accumulation. After cellular internalization, the PCP coating stimulatingly dissociates exposing to the high‐level ROS, and releases ES and Cu, thereby triggering cell death via cuproptosis. Meanwhile, Cu^2+^‐stimulated Fenton‐like reaction together with CA‐stimulated ROS production simultaneously breaks the redox homeostasis, which compensates for the insufficient oxidative stress treated with ES alone, in turn inducing immunogenic cell death of tumor cells, achieving simultaneous cuproptosis and immunotherapy. Furthermore, the excessive ROS accelerates the stimuli‐dissociation of ECPCP, forming a positive feedback therapy loop against tumor self‐alleviation. Therefore, ECPCP as a nanoplatform for cuproptosis and immunotherapy improves the dual antitumor mechanism of ES and provides a potential optimization for ES clinical application.

## Graphical abstract

In this study, a self‐ampslifying ROS‐responsive nanoplatform (ECPCP) is developed, of which a polymer based on cinnamaldehyde and polyethylene glycol is fabricated to encapsulate elesclomol‐Cu compound. ECPCP successfully prolongs the systemic circulation of elesclomol and enhances its tumor accumulation, subsequently improving the dual antitumor mechanism of ES, cuproptosis, and immunotherapy, and provides a potential optimization for ES clinical application.

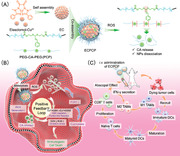

## 1 Introduction

Cuproptosis, a unique form of cell death, has been elucidated recently, which is distinct from other well‐known cell death pathways such as necroptosis, apoptosis, and ferroptosis.^[^ ^1^, ^2^, ^3^ ^]^ It is initiated by a copper overload and lipoylated components of the tricarboxylic acid cycle, leading to lipoylated protein aggregation and the disappearance of Fe‐S cluster proteins, thereby resulting in proteotoxic stress for cell death.^[^ ^4^, ^5^, ^6^ ^]^ Cuproptosis is a copper‐dependent pathway, and its efficiency is heavily determined by the intracellular copper concentration. However, intracellular copper is regulated at an extraordinarily low level through concentration‐sensitive homeostatic mechanisms to prevent the overload of free copper that is harmful to cells.^[^ ^6^, ^7^ ^]^ To this end, copper ionophores are utilized to disequilibrate the copper balance and wildly transport copper into tumor cells.^[^ ^8^, ^9^ ^]^ At present, many copper ionophores have been applied for cuproptosis, such as disulfiram (DSF), 8‐hydroxyquinolines and elesclomol (ES), etc.^[^ ^10^ ^]^ Among them, ES is considered the most attractive due to its extremely high transfer rate and the ability to selectively transport Cu into mitochondria.^[^ ^11^, ^12^ ^]^ However, when applied clinically, ES is criticized for rapidly removing and metabolizing in the circulation, resulting in a short half‐life and limited tumor accumulation. Thus, improving the cycling stability and tumor targeting of ES is an urgent need.

In addition to the emerging cuproptosis, the initial research and development significance of ES can't be ignored. Because intracellular Cu^2+^ can be oxidized to Cu^+^ and generate ROS via Fenton‐like reaction, ultimately inducing apoptosis for tumor death.^[^ ^13^, ^14^, ^15^, ^16^ ^]^ Furthermore, intracellular ROS generation can also trigger oxidative stress, which can subsequently result in immunogenic cell death (ICD) for cancer immunotherapy.^[^ ^17^ ^]^ Dying tumor cells under ICD can induce calreticulin (CRT) expression, high mobility group protein (HMGB1) secretion, and adenosine triphosphate (ATP) release, which can stimulate dendritic cell maturation and antitumor immune response.^[^ ^18^ ^]^ ES was introduced into clinical research due to its excellent Cu transport capability. The outcomes indicated that ES owed satisfactory biosafety, benefiting from the low basal ROS level of normal cells. However, its antitumor results were not good enough to be approved.^[^ ^19^ ^]^ Apart from the reasons mentioned above, the strong self‐alleviation ability of tumor cells against oxidative stress also reduces the efficacy of ES,^[^ ^8^, ^12^ ^]^ for which the ROS production by Cu‐mediated Fenton‐like reaction of ES may not achieve the expectant functions. Therefore, increasing intracellular Cu accumulation together with breaking redox homeostasis may be a valuable strategy to promote the dual antitumor mechanism of ES.

**Scheme 1:**
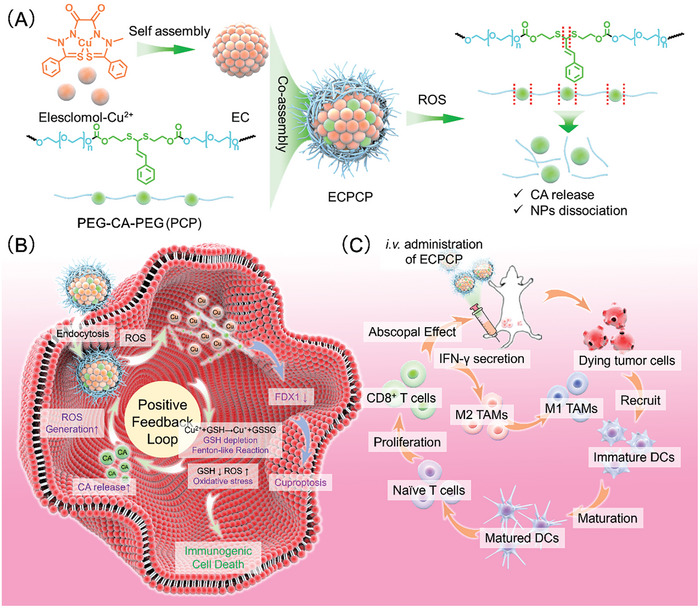
Schematic illustration of the preparation and mechanism of ECPCP for cuproptosis‐ICD combination therapy.

Herein, cinnamaldehyde (CA), a major component of cinnamon, is selected for co‐therapy with ES. CA is an FDA‐approved natural food additive and pharmaceutical ingredient, which has strong anticancer activity due to its mitochondrial dysfunction capability. It can selectively induce oxidative stress with massive ROS production and decrease mitochondrial membrane potential, subsequently inducing procaspase processing for tumor killing.^[^ ^20^, ^21^, ^22^ ^]^ While, CA displays unsatisfied solubility and tumor targetability, as well as poor bioavailability due to the high toxicity.^[^ ^23^, ^24^ ^]^ To co‐delivery ES and CA, an amphiphilic polymer (PCP) with ROS‐sensitive thioketal bonds was first synthesized and then applied to encapsulate ES and Cu compound (EC), forming ECPCP (**Scheme** **1**). ECPCP could effectively accumulate in tumor tissues via enhanced permeability and retention EPR effect (EPR). After cellular internalization, the PCP coating stimulatingly degraded when exposed to the high‐level ROS and released CA and EC. Subsequently, EC dissociated into ES and Cu. On one side, the released Cu^2+^ could bind to ferredoxin 1 (FDX1) and be reduced to Cu^+^, while ES could chelate and transport extracellular Cu^2+^ into tumor cells, resulting in a continuous Cu accumulation to induce cuproptosis.^[^ ^25^ ^]^ On the other side, Cu^2+^ could react with GSH and transform into highly toxic Cu^+^ and GSSG. Then, Cu^+^ triggered the Fenton‐like reaction, which together with CA‐induced oxidative stress in turn facilitated CA release and ROS production, finally forming a positive feedback antitumor loop. Notably, ECPCP with the continuous ROS generation triggered ICD of tumor cells, promoted the recruitment and maturation of dendritic cells, and improved T cell infiltration, greatly enhancing the immunogenicity of tumor cells.^[^ ^26^, ^27^, ^28^ ^]^ Naturally, ECPCP achieved excellent antitumor efficacy with the optimized dual‐function, cuproptosis and cancer immunotherapy, and is considered as an attractive strategy for clinical application of ES.

## 2 Results and Discussion

### 2.1 Preparation and Characterization of ECPCP

EC was fabricated via both coordination force and π–π stacking.^[^ ^11^ ^]^ PCP was synthesized with a ROS‐responsive CA‐based thioketal linkage and carboxylation‐PEG (Figures S1 and S2, Supporting Information), which could release CA exposing to the high‐level intracellular ROS of tumor cells. Subsequently, EC was encapsulated by PCP via electrostatic and hydrophobic interaction, forming ECPCP. Meanwhile, PNP, negative control without ROS‐responsive CA release characteristic, was also synthesized (Figures S3 and S4, Supporting Information). The cytotoxicity of both PCP and PNP was investigated. As shown in Figure S5 (Supporting Information), PNP displayed almost no effect on the viability against 4T1 cell line, while PCP reflected concentration‐dependent cell cytotoxicity. The cytotoxicity was mainly ascribed to the released CA, which could significantly aggravate oxidative stress.^[^ ^22^ ^]^

**Figure 1:**
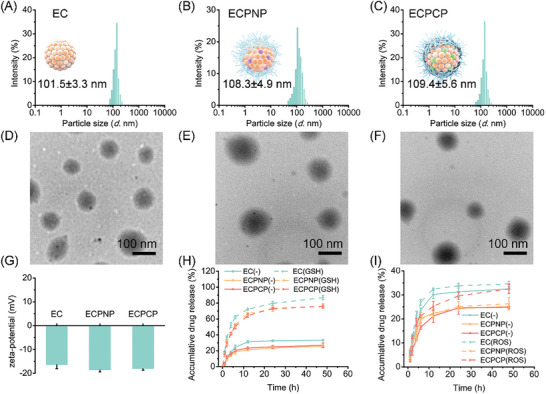
Characterization of ECPCP. A–C) Particle size distribution of EC, ECPNP, and ECPCP. D–F) TEM images of EC, ECPNP, and ECPCP. Scale bar: 100 nm. G) Zeta‐potential of EC, ECPNP, and ECPCP. H) In vitro ES release of EC, ECPNP, and ECPCP under GSH condition (GSH concentration: 10 mm). I) In vitro ES release of EC, ECPNP, and ECPCP under ROS condition (H_2_O_2_ concentration: 50 µm). Data are presented as mean ± SD, *n* = 3.

The physicochemical characteristics of the nanoparticles were fully investigated. The particle size of EC, ECPNP and ECPCP was measured by DLS, and the diameter was around 101.5, 108.3, and 109.4 nm Figure (**1**A**–**C), respectively. The morphology was observed by transmission electrostatic microscopy (TEM). As shown in Figure 1D–F, the nanoparticles were all displayed homogeneous spheroid‐like structures. Zeta‐potential was also measured, as ‐16.5, ‐18.6, and ‐18.1 mV for EC, ECPNP, and ECPCP (Figure 1G), respectively. Then, the in vitro stability of the nanoparticles was confirmed. As shown in Figure S6 (Supporting Information), EC tended to aggregate in both PBS and DMEM, which was attributed to the coordination force between ES and Cu^2+^. On the contrary, ECPNP and ECPCP reflected favorable stability during 7‐day incubation with no obvious particle size alteration, suggesting that the PCP or PNP layer could effectively coat the EC core and avoid EC aggregation with the PEG segment. Commonly, nanoparticles with appropriate and stable particle sizes are considered more likely to undergo systemic circulation and tumor accumulation.

### 2.2 In Vitro Release of ECPCP

Suitable controlled release of antitumor active ingredient is meaningful and even largely determines the balance of efficacy and biocompatibility. It has been fully investigated that compared with normal cells, tumor environment presents characteristics such as high ROS and GSH levels owing to abnormal metabolism by rapid cell proliferation.^[^ ^29^, ^30^, ^31^ ^]^ Therefore, condition mediums were customized to simulate the tumor environments for evaluating the drug release behavior of the nanoparticles, including neutral condition, ROS condition (H_2_O_2_ concentration: 50 µm), and GSH condition (GSH concentration: 10 mm). First, CA release from ECPCP and ECPNP was measured. As shown in Figure S7 (Supporting Information), <5% of CA was released from both ECPCP and ECPNP during 24 h under the neutral condition, implying that both PCP and PNP polymers displayed a satisfied stability. However, with extra H_2_O_2_, CA release from ECPCP was significantly accelerated (≈86.7%), while ECPNP was not affected. The results confirmed the ROS‐responsive characteristics of PCP polymer and implied that ECPCP could selectively release CA at the tumor site.

Although ES is known for its high biosafety, some studies have reported that excessive doses of ES are still toxic.^[^ ^32^ ^]^ Thus, we are also interested in providing ES‐responsive release for reducing the initial dosage without prejudicing its tumor accumulation. As shown in Figure 1H, in the neutral condition, EC, ECPNP, and ECPCP showed a sustained ES release. Around 30% ES was released from the carriers during 48 h. Compared with the EC group, ECPNP and ECPCP displayed a relatively low release rate, because the polymer layers formed a hydrophilic shell and prevented the diffusion of ES from the EC core. Subsequently, the drug release was performed with 10 mm GSH. In stark contrast, significant ES release elevation was observed in all the groups, as which ≈80% of ES escaped from EC during 48 h. It has been reported that Cu^2+^ could effectively react with GSH to produce Cu^+^ and GSSG.^[^ ^33^, ^34^, ^35^ ^]^ Therefore, we hypothesized that this phenomenon was due to the higher affinity between Cu^2+^ and GSH, which was more tendentious to induce the dissociation of EC and facilitate ES release. In addition, ES release under the ROS condition was also measured. As shown in Figure 1I, there was no obvious difference for EC or ECPNP groups between neutral and ROS conditions. However, the ES release rate of ECPCP was obviously accelerated with the extra H_2_O_2_, and the accumulative release was comparable to that of the EC group after 24 h, benefiting from the dissociation of PCP polymer under ROS condition. Herein, we believed that ECPCP displayed ROS/GSH dual‐responsive characteristics, which could stimuli‐release ES in the tumor site with reduced systematic leakage and toxicity.

In addition, we speculate that there may be a positive feedback release process when ECPCP is applied to tumor cells (Figure 1H,I; Figure S7, Supporting Information), in which the stimuli‐release CA and ES could induce ROS accumulation and further accelerate the intracellular release of ECPCP. These may lead to enhanced instantaneous concentration of CA and ES within tumor cells. Considering the repulsive self‐alleviation ability and adaptability of tumor cells, ECPCP may be praiseworthy as the positive feedback release process.

### 2.3 In Vitro Antitumor Activity

**Figure 2:**
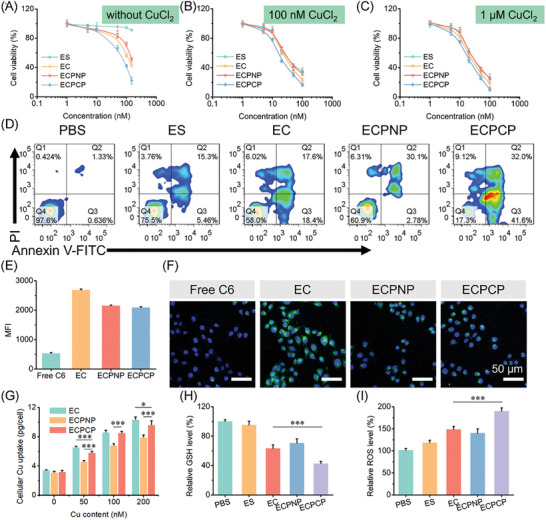
In vitro antitumor activity of ECPCP. A–C) In vitro cytotoxicity of the nanoparticles against 4T1 cell line without CuCl_2_, with 100 nm CuCl_2_ and 1 µm CuCl_2_, respectively. D) Representative FCM profiles of the apoptosis percentage of 4T1 cell line. E,F) FCM and CLSM observation of the cellular uptake of C6 labeled nanoparticles, respectively. Scale bar: 50 µm. G) Intracellular Cu uptake assay. H,I) Intracellular GSH and ROS level assay. Data are presented as mean ± SD, *n* = 6. “*”, “**” and “***” represented *p* < 0.05, *p* < 0.01, and *p* < 0.001, respectively.

ES is a recognized Cu ionophore and expert in transporting Cu into mitochondria, thus potentially killing tumor cells via cuproptosis pathway.^[^ ^8^ ^]^ First, we explored the antitumor activity of the nanoparticles. MTT assay was applied with 4T1 cell line. As shown in **Figure** **2**A, the IC_50_ value of ES was more than 200 nm, indicating that ES had almost no cytotoxicity. While, the antitumor capacity of the preparations was improved by loading Cu^2+^. The IC_50_ value was 128.1, 169.7, and 74.75 nM for EC, ECPNP, and ECPCP, respectively. Furthermore, we also investigated the antitumor efficacy of the nanoparticles in the presence of CuCl_2_. The cytotoxicity of CuCl_2_ was first validated. As shown in Figure S8 (Supporting Information), it had almost no effect even with the concentration of CuCl_2_ reaching 1 µm. After pre‐incubation with 100 nm CuCl_2_, the IC_50_ value of ES, EC, ECPNP, and ECPCP against 4T1 cell line were 44.54, 33.96, 42.12, and 23.95 nm, respectively, which were significantly lower than that without CuCl_2_ (Figure 2B). Moreover, when the concentration of CuCl_2_ reached 1 µm, IC_50_ value of the nanoparticles was further decreased (Figure 2C), possessing a concentration‐dependent characteristic. The result suggested that EC, ECPNP, and ECPCP could all transport Cu into cytoplasm and displayed a Cu‐dependent antitumor activity. Notably, compared with ES, EC, and ECPNP groups, ECPCP obtained the highest cytotoxicity regardless of CuCl_2_ pre‐incubation. This was mainly attributed to the ROS responsive‐release CA, which aggravated the oxidative stress by ROS generation and amplified the antitumor efficacy of cuproptosis by reducing the GSH level.^[^ ^6^ ^]^

Then, Annexin V‐FITC/PI assay was applied to evaluate the cytotoxicity of the nanoparticles. As shown in Figure 2D, the apoptosis percentage of ECPCP‐treated cells was 73.6%, which was 3.55, 2.04, and 2.24 times higher than that of ES, EC, and ECPNP, respectively. Taken together, ECPCP was considered to kill the tumor cells via a simultaneous effect.

### 2.4 In Vitro Cellular Uptake Assay

Then, we explored the antitumor mechanism of the nanoparticles. As cytotoxicity is commonly related to the concentration of intercellular toxic components, cellular uptake was investigated. Coumarin 6 (C6) was selected as the fluorescence probe for flow cytometry assay and CLSM assay. As shown in Figure 2E,F, EC unveiled the highest cellular uptake compared with ECPNP and ECPCP. This was due to the PEG segment of PNP and PCP shells, which could prevent the interaction between cells and nanoparticles.^[^ ^36^ ^]^ Despite being affected by PEG shells, ECPCP and ECPNP could also increase the cellular uptake capability compared with free C6 against 4T1 cells.

In addition, intracellular Cu concentration was also measured to evaluate the Cu transport efficiency. As shown in Figure 2G, all the nanoparticles displayed Cu‐dependent transport characteristics. Among them, EC rightfully performed best due to the excellent cellular uptake. While, ECPCP could also effectively transport Cu into 4T1 cells. This phenomenon was probably because the Cu transport was positively correlated to the concentration of free ES, and ECPCP did well in ES release with the ROS‐responsive dissociated shell. For the same reason, ECPNP with the undissociated layer deservedly gained less Cu accumulation (Figure 2G) and antitumor activity (Figure 2A–C).

### 2.5 GSH and ROS Level Measurement

Tumor cells are characterized as high redox balance, and breaking the homeostasis has been proven to be a mature therapeutic strategy.^[^ ^18^ ^]^ Herein, the intercellular GSH and ROS were detected. As shown in Figure 2H,I, all Cu‐based nanoparticles could effectively reduce intracellular GSH and enhance the ROS level. Generally, the oxidative stress increases with the concentration of Cu.^[^ ^7^, ^37^ ^]^ After interaction with GSH, Cu^2+^ was transformed to Cu^+^ and reacted with H_2_O_2_ to produce highly toxic ·OH which was also involved in the process of ROS production.^[^ ^33^ ^]^ However, in this study, ECPCP performed best among the nanoparticles, which was inconsistent with the Cu concentration in the tumor cells (Figure 2G). This was considered as the simultaneous effect of both Cu and dissociated CA. Cu‐induced GSH depletion and CA‐activated ROS production simultaneously broke the intracellular redox homeostasis and enlarged the oxidative stress.

### 2.6 Cuproptosis‐Related Assay

**Figure 3:**
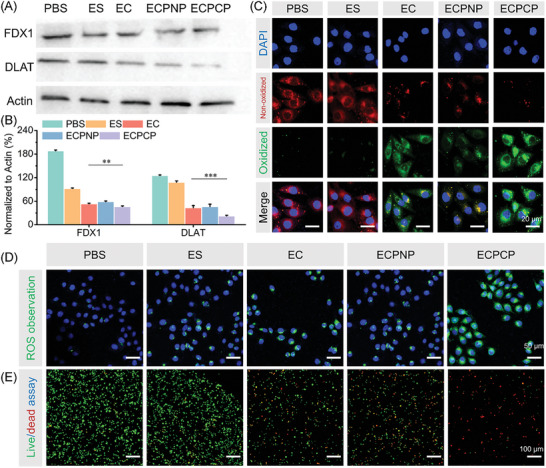
In vitro validation of cuproptosis and oxidative stress. A) FDX1 and DLAT expression measured by western blotting. B) Semi‐quantitative analysis of FDX1 and DLAT expression. C) LPO accumulation in 4T1 cells after treatment detected by CLSM. Scale bar: 20 µm. D) Intracellular ROS level after treatment visualized by DCFH‐DA. Scale bar: 50 µm. E) Live/dead assay. Green and red fluorescence represented live and dead cells, respectively. Scale bar: 100 µm. Data are presented as mean ± SD, *n* = 3. “*”, “**” and “***” represented *p* < 0.05, *p* < 0.01, and *p* < 0.001, respectively.

Then, we validated the cuproptosis of the nanoparticles. During cuproptosis pathway, factors such as FDX1 could regulate several enzymes including dihydrolipoamide S‐acetyltransferase (DLAT) to undergo lipoylation.^[^ ^8^ ^]^ FDX1 and DLAT expression as hallmarks of cuproptosis were both measured. As shown in Figure **3**A,B, EC, ECPNP, and ECPCP could effectively down‐regulate FDX1 and DLAT expression, especially for ECPNP, indicating that the nanoparticles could induce cell death via cuproptosis.

Redox homeostasis break was also confirmed by lipid peroxidation (LPO) level detection by CLSM.^[^ ^38^ ^]^ C11BODIPY was used as a fluorescence probe which could produce green fluorescence in the presence of LPO. As shown in Figure 3C, almost no green fluorescence was observed in PBS and ES groups, while EC, ECPNP, and ECPCP displayed obvious fluorescence, indicating that nanoparticles with Cu could induce LPO accumulation. Moreover, compared with ECPNP and EC groups, ECPCP gained the strongest fluorescence intensity, suggesting that ECPCP successfully broke redox homeostasis and increased intracellular ROS to facilitate LPO production. Then, the intracellular ROS level was detected with DCFH‐DA which could be oxidized by ROS and produce DCFH with green fluorescence. As shown in Figure 3D, the optimum ROS production capability of the ECPCP group was proven by the brightest green fluorescence.

Cuproptosis together with ROS production will effectively induce cell death. Therefore, a live/dead assay was also investigated. As shown in Figure 3E, the results revealed that the largest number of dead cells was found for ECPCP, further proving the combination effect of cuproptosis and amplified oxidative stress.

### 2.7 ICD Induced by ECPCP

**Figure 4:**
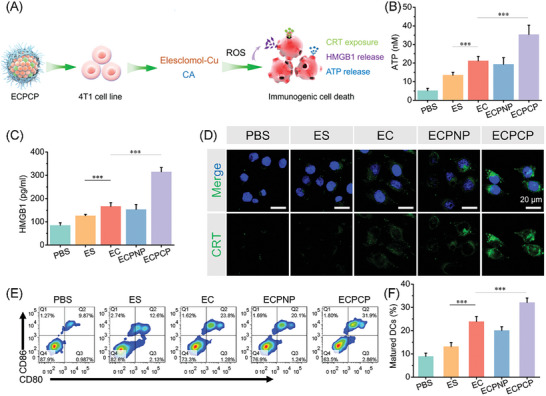
In vitro ICD induction by ECPCP. A) Schematic illustration of ICD induced by ECPCP. B) Extracellular ATP content measurement after treatment. C) HMGB1 secretion by 4T1 cell line. D) CRT exposure observation by CLSM. Green and blue fluorescence represented CRT and nucleic, respectively. Scale bar: 20 µm. E,F) FCM image and histogram analysis of matured DCs percentages. Data are presented as mean ± SD, *n* = 6. “*”, “**” and “***” represented *p* < 0.05, *p* < 0.01, and *p* < 0.001, respectively.

High intracellular ROS can act as a signal to induce tumor cell death via ICD.^[^ ^39^ ^]^ The results above demonstrated that ECPCP could effectively increase ROS production and break oxidative/redox balance. Therefore, we are interested in investigating whether ECPCP could induce ICD and activate antitumor immune response. The possible mechanism of ICD induced by ECPCP is shown in **Figure** **4**A. Tumor cells undergoing ICD can increase CRT expression, HMGB1 secretion, and ATP release, which is known as damage‐associated molecular patterns (DAMPs). DMAPs act as “eat me” signals to increase dendritic cell maturation and antigen‐presenting for activating antitumor immunity.^[^ ^40^ ^]^ ICD‐related hallmarks were measured in this work.

Notably, significant elevation of ATP release (Figure 4B) and HMGB1 secretion (Figure 4C) was found for 4T1 cells treated with ECPCP. Meanwhile, the brightest green fluorescence (presented as CRT) distributed around the nucleus when treated with ECPCP (Figure 4D). The results confirmed that ECPCP could effectively induce tumor cell killing via ICD pathway. Subsequently, DC maturation was also investigated. As shown in Figure 4E,F, the percentage of matured DCs for the ECPCP group was 2.43 times, 1.34 times, and 1.59 times higher than that of ES, EC, and ECPNP groups. All these valuable results proved our hypothesis that ECPCP mediated‐combination therapy could effectively induce ICD to release DAMPs, thereby increasing the immunogenicity of tumor cells and activating antitumor immune response.

### 2.8 In Vivo Biodistribution of ECPCP

**Figure 5:**
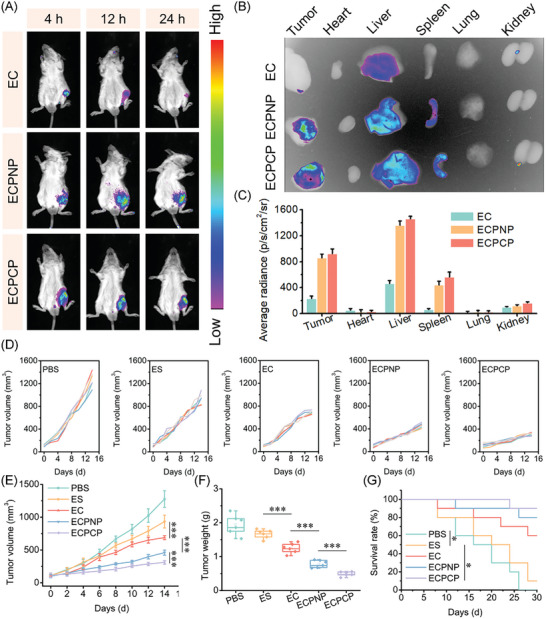
In vivo antitumor activity of ECPCP. A) In vivo biodistribution of the nanoparticles against tumor‐bearing mice model during 24 h. B,C) Ex vivo biodistribution and semi‐quantitative analysis of the nanoparticles in major organs at 24 h time point, *n* = 3. D) Individual tumor volume curve treated with the nanoparticles for 14 days. E) Average tumor volume curve treated with the nanoparticles. F) Tumor weight on 14th day. G) Survival curve of the mice after treatment. Data are presented as mean ± SD, *n* = 6. “*”, “**” and “***” represented *p* < 0.05, *p* < 0.01, and *p* < 0.001, respectively.

The in vivo biodistribution and tumor accumulation of antitumor‐active ingredients greatly affect the therapeutic efficacy. To investigate the in vivo biodistribution of the nanoparticles, a subcutaneous tumor model was established based on 4T1 cells. Tumor‐bearing mice were received intravenous administrated with DiR‐labeled nanoparticles, and the in vivo biodistribution was evaluated by IVIS. As shown in **Figure** **5**A, the nanoparticles owed favorable tumor targetability due to the EPR effect. For the EC group, a strong fluorescence signal was observed at 4 h and gradually decreased until 24 h. In comparison, both ECPNP and ECPCP displayed a long‐lasting tumor accumulation, implying that PEG‐based polymer modified nanoparticles effectively enhanced in vivo circulation time and gained long retention at tumor site.

At 24 h time point, the mice were sacrificed and major organs were harvested for ex vivo biodistribution. As shown in Figure 5B,C, for the EC group, a limited fluorescence signal was found in tumor tissues and most of the EC was accumulated in the liver, suggesting that EC was quickly metabolized. Notably, there was an obvious fluorescence signal for both ECPCP and ECPNP in tumor sites, and the fluorescence signal in the liver and spleen was also stronger compared with the EC group. All these results indicated that ECPCP could effectively target and accumulate in tumor sites. Herein, we speculated that ECPCP could improve the half‐time of ES and potentially satisfied for clinical application.

### 2.9 In Vivo Antitumor Activity of ECPCP

Given the above results, ECPCP was concluded to be an excellent tumor killer in vitro. To validate the antitumor efficacy in vivo, a subcutaneous tumor mice model was established, and the mice were randomized and treated with PBS, ES, EC, ECPNP and ECPCP, respectively. Tumor volume during the treatment was recorded and analyzed (Figure 5D,E), and the tumor was weighed on the 14^th^ day (Figure 5F). ES displayed a limited tumor inhibition effect on 14th day (inhibition rate: 14.5%), which was mainly attributed to the unsatisfied tumor accumulation of free ES. Benefiting from the EPR effect, EC gained higher antitumor efficacy (inhibition rate: 36.1%) compared with ES. After modification with PEG‐based polymers, both ECPCP and ECPNP showed optimized antitumor activity with enhanced in vivo circulation time and tumor retention, while ECPCP performed best (inhibition rate: 74.8%) among all the reference groups. The survival rate was also investigated (Figure 5G). Similarly, ECPCP could effectively enhance the survival time of tumor‐bearing mice. All these results demonstrated that ECPCP had the most superior antitumor activity with effective cuproptosis‐inducing effect and self‐amplifying ROS production capability.

**Figure 6:**
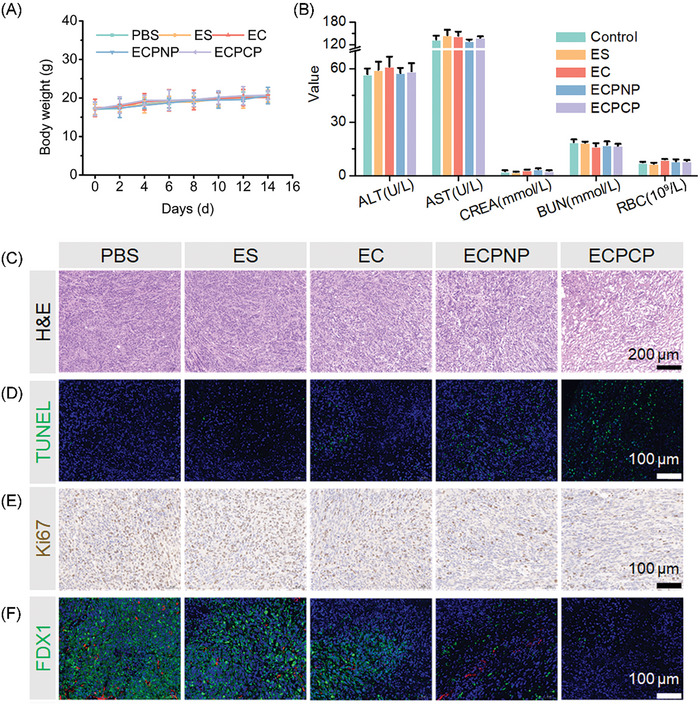
Biosafety and histological assay of ECPCP. A) Body weight of the mice during the treatment, *n* = 6. B) Mice blood analysis, *n* = 4. C–E) H&E staining, TUNEL immunofluorescence, Ki67 immunohistochemistry of the tumor tissues, respectively. F) Validation of cuproptosis by FDX1. Green fluorescence: marker, red fluorescence: blood vessel. Data are presented as mean ± SD.

Next, we evaluated the biosafety of the nanoparticles. The body weight of the mice was measured during the treatment, and there were no statistical differences among the groups (**Figure** **6**A). Meanwhile, the blood samples of the mice were collected for serum biochemistry assay. No obvious change in alanine aminotransferase (ALT), aspartate aminotransferase (AST), creatinine (CREA), blood urea nitrogen (BUN), and red blood cell counts (RBC) was observed (Figure 6B). Furthermore, the major organs were harvested for H&E staining. As shown in Figure S9 (Supporting Information), ECPCP had no tissue toxicity. It indicated that ECPCP displayed an applicable biosafety and was suitable for intravenous administration.

Finally, the tumor tissues were obtained for histological assay. H&E staining and TUNEL immunofluorescence showed that ECPCP effectively induced nuclear fragmentation, nucleolysis as well as tumor cell apoptosis (Figure 6C,D). Ki67 assay was also performed. As shown in Figure 6E, ECPCP could significantly inhibit tumor proliferation.

### 2.10 In Vivo Cuproptosis and Antitumor Immune Response Analysis

We further investigated whether the nanoparticles could induce cuproptosis and activate antitumor immunity in vivo. Cuproptosis heavily relies on intracellular Cu content, which can be oxidized to form highly toxic monovalent copper by FDX1 and then regulate DLAT to undergo lipoylation, which will induce proteotoxic stress for cell death. Therefore, cuproptosis‐related marker FDX1 expression was measured by immunofluorescence. Green and red fluorescence represented marker and blood vessel, respectively. As shown in Figure 6F, ECPCP could effectively inhibit the expression of FDX1. Furthermore, FDX1 expression was also detected by western blot (Figure S10, Supporting Information), and the results were in a similar trend with the immunofluorescence assay. All these abovementioned results implied that ECPCP had a strong capability to induce tumor cell death via cuproptosis pathway.

**Figure 7:**
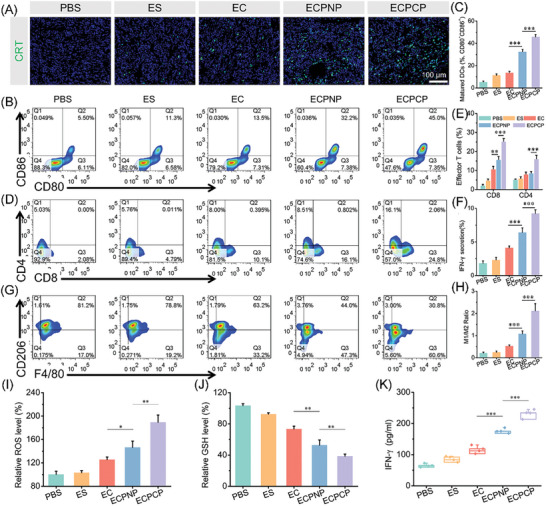
In vivo validation of antitumor immune response of ECPCP. A) CRT immunofluorescence of tumor tissues. Scale bar: 100 µm. B) Flow cytometry analysis of matured DCs in TDLNs. C) Histogram analysis of the percentages of matured DCs. D) Effector T lymphocytes in tumor tissues. E) Histogram analysis of the effector T lymphocyte percentages. F) IFN‐γ secretion percentages of CD8^+^ T cells. G) M2‐TAMs in tumor tissues. H) M1/M2 ratio in tumor tissues. I–K) In vivo ROS, GSH, and IFN‐γ levels in tumor tissues, respectively. Data are presented as mean ± SD, *n* = 4. “*”, “**” and “***” represented *p* < 0.05, *p* < 0.01, and *p* < 0.001, respectively.

CRT, a hallmark of ICD that could serve as an “eat me” signal for DC recruitment, was investigated by immunofluorescence assay. Green fluorescence indicated CRT‐positive cells. As shown in **Figure** **7**A, ECPCP gained the strongest fluorescence intensity and CRT exposure among the groups. The results confirmed that ECPCP could effectively activate antitumor immune response via ICD pathway. Then, matured DC percentages in tumor‐draining lymph nodes (TDLNs) were measured and gated as CD80^+^CD86^+^. As shown in Figure 7B,C, 45.8% of DCs were found to be matured for mice treated with ECPCP, which was significantly higher than that treated with other nanoparticles, suggesting that ECPCP effectively induced DCs maturation for antigen‐presenting. Efficient antigen‐presenting capability by DCs will stimulate naïve T cell proliferation and infiltration into tumor tissues for effective tumor killing. Subsequently, T‐cell infiltration was analyzed. The results showed that ECPCP obviously increased CD4^+^ and CD8^+^ T cell infiltration in tumor tissues for cancer immunotherapy (Figure 7D,E). It is well known that tumor‐killing capability of CD8^+^ T cells is heavily relied on IFN‐γ production.^[^ ^41^ ^]^ Therefore, IFN‐γ secretion from CD8^+^ T cells was measured, and prominent IFN‐γ secretion elevation was found for the ECPCP group (Figure 7F; Figure S11, Supporting Information). Of note, EC could cause cuproptosis and ICD, which active antitumor immune response. However, low tumor accumulation capability and limited intracellular internalization hampered its antitumor activity. Meanwhile, a strong antioxidant system of tumor cells would also restrict its efficiency. In comparison, ECPCP displayed stronger antitumor activity and immune response, which was mainly ascribed to the additional ROS production capability of PCP polymer. Subsequently, continuous ROS generation could effectively break the redox homeostasis of tumor cells and induce cell death via ICD pathway. Therefore, ECPCP would display a combinational effect for tumor killing.

It is reported that 50% of tumor mass are tumor‐associated macrophages (TAMs), which are surrounded and connected with tumor cells for tumor proliferation, extracellular matrix remodeling, and tumor immune tolerance.^[^ ^42^ ^]^ TAMs are mainly divided into pro‐inflammatory M1‐type and anti‐inflammatory M2‐type. In tumor tissues, M2‐TAMs are widely distributed, inhibiting the proliferation of T cells and inducing tumor immune escape.^[^ ^43^ ^]^ Therefore, we investigated the polarization of TAMs in tumor tissues. F4/80^+^CD206^+^ represented M2‐TAMs and F4/80^+^CD206^−^ represented M1‐TAMs. As shown in Figure 7G,H, a significant elevation of the M1/M2 ratio was found when treated with ECPCP, and the ratio was also enhanced in EC and ECPNP groups. Meanwhile, the enhanced effector T cell infiltration would cause a large amount of pro‐inflammatory cytokines secretion, which in turn induce an inflammatory response and polarize macrophages into M1 phenotype. To sum up, we considered that cuproptosis and ICD induced by ECPCP could effectively reduce tumor cell proliferation and overcome the immunosuppressive microenvironment of solid tumors.

In vivo ROS and GSH levels were also detected. As shown in Figure 7I,J, ROS was remarkably increased in tumors of mice treated with ECPCP, and the GSH level was decreased. The results confirmed that the redox homeostasis was destroyed in vivo. Finally, IFN‐γ and TNF‐α level in tumor tissues was measured by ELISA kit. As shown in Figure 7K and Figure S12 (Supporting Information), ECPCP induced IFN‐γ and TNF‐α production, indicating activated antitumor immunity.

### 2.11 Bilateral Antitumor Activity by ECPCP

**Figure 8:**
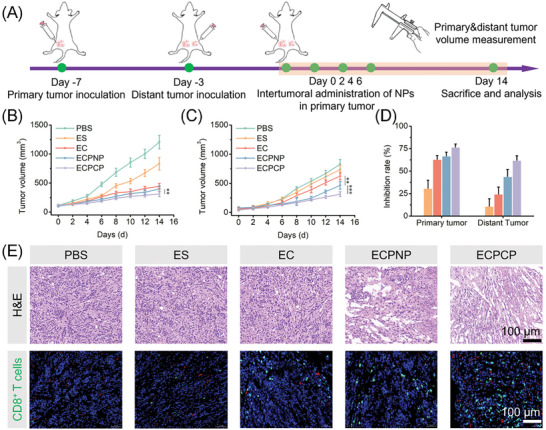
A) Establishment of bilateral tumor model and the timeline of administration. B,C) Average tumor volume of primary tumor and distant tumor, respectively, *n* = 6. D) Inhibition rate of primary tumor and distant tumor. E) H&E staining and CD8^+^ T cells immunofluorescence staining of distant tumor tissues. Data are presented as mean ± SD. “*”, “**” and “***” represented *p* < 0.05, *p* < 0.01, and *p* < 0.001, respectively.

To further demonstrate whether the activated antitumor immune response by ECPCP was functional, we established a bilateral model of 4T1 cell. The timeline of the study is illustrated in **Figure** **8**A. The nanoparticles were intertumoral injected into the primary tumor, and the tumor volume of both primary and distant tumors was monitored (Figure 8B,C). The inhibition rate was also calculated. Compared with the intravenous administration assay (Figure 5B), better antitumor activity was found in all the groups for intertumoral administration (Figure 8D), which was due to the optimized tumor accumulation and retention of the nanoparticles. Among the primary groups, ECPCP naturally displayed the strongest antitumor efficacy. At the same time, ECPCP also gained the highest inhibition rate against distant tumors with activated antitumor immune response. Finally, the distant tumors were harvested for H&E staining and CD8^+^ T cell immunofluorescence. As shown in Figure 8E, ECPCP could effectively induce nucleic fragmentation and cytoplasm loss as well as CD8^+^ T cell infiltration. The encouraging results confirmed the activated antitumor immune response of ECPCP.

## 3 Conclusion

In this study, a novel ROS‐responsive self‐amplifying nanoplatform was fabricated to co‐delivery ES and CA for cuproptosis and cancer immunotherapy. It showed that ECPCP significantly improved the circulation and tumor accumulation of ES. After cellular uptake by tumor cells, ECPCP rapidly degraded in a positive feedback manner and released CA and EC, and then EC further dissociated into ES and Cu. The released Cu^2+^ not only induced cuproptosis cell death, but also mediated ICD together with CA and activated immune response. In summary, ECPCP promoted the dual antitumor mechanism of ES and gained excellent antitumor efficacy, which is considered a potential optimization for ES clinical application. However, there are still many limitations to this drug delivery system. First, long‐term tissue toxicity and systematic pharmacokinetics of ECPCP need to be further explored. Moreover, the cuproptosis‐immunotherapy strategy was only validated against 4T1 tumor model and a wide variety of tumor cell lines should be further investigated. More investigations should be also investigated about ECPCP, such as administration in combination with immune checkpoint inhibitors or other immunotherapeutic agents.

## 4 Experimental Section

### Chemicals and Reagents

Elesclomol (ES), cinnamaldehyde (CA), mercaptoethanol, 2‐amino‐1,3‐propanediol 4‐dimethylaminopyridine (DMAP), 1‐(3‐dimethylaminopropyl)‐3‐ethylcarbodiimide hydrochloride (EDC), and coumarin 6 (C6) were all purchased from Aladdin Biotechnology (Shanghai, China). COOH‐PEG_2k_‐COOH was bought from Weihua Biotechnology (Guangdong, China). 1,1'‐dioctadecyl‐3,3,3',3'‐tetramethylindotricarbocyanine iodide (DiR), 3‐(4,5‐dimethylthiazol‐2‐yl)‐2,5‐diphenyltetrazolium bromide (MTT), Annexin V‐FITC/PI kit, live/dead kit, 2‐(4‐aminophenyl)‐1H‐indole‐6‐carboxamidine (DAPI), dichlorodihydrofluorescein diacetate (DCFH‐DA), ROS detection kit, culture medium and fetal bovine serum (FBS) were all purchased from Servicebio (Wuhan, China). Luminescent ATP detection assay kit and anti‐calreticulin antibody were all purchased from Abcam (USA). GSH detection kit was purchased from KeyGen BioTECH (Nanjing, China). Copper (Cu) assay kit was purchased from Jiancheng Bioengineering Institute (Nanjing, China). All fluorescence‐labeled antibodies for immune cell analysis were supplied from Aifang Biological (Changsha, China).

### Cells and Animals

Murine 4T1 cell line was purchased from Meilun Biotechnology Company (Dalian, China) and supplemented with 10% FBS at 37 °C with 5% CO_2_. Bone marrow‐derived dendritic cells were isolated from the tibia of Balb/c mice and polarized with GM‐CSF/IL‐4 according to the reference.

For 4T1 tumor‐bearing Balb/c mice model establishing, 1×10^7^ cells were suspended into 0.2 ml PBS and subcutaneously administrated into the buttocks of mice. When the tumor volume reached about 100 mm^3^, the mice were randomized in groups for further studies. All animal experiments were approved by the Institutional Animal Care and Use Committee (IACUC) of China Pharmaceutical University.

### Synthesis of ROS‐Responsive CA‐Based Linker

The synthesis of CROH, a ROS‐responsive CA‐based linker, was displayed in Figure S1A (Supporting Information) according to the literature.^[^ ^44^ ^]^ First, CA and mercaptoethanol were co‐dissolved in ethyl acetate with hydrochloric acid as a catalyst. The mixture was reacted with magnetic stirring under a nitrogen atmosphere (4 h, 25 °C). Subsequently, the organic solvent was removed, and the crude product was purified by silica gel column chromatography to get CROH.

The negative control CA‐based linker without ROS‐responsive characteristic was synthesized as displayed in Figure S3A (Supporting Information). Briefly, CA and 2‐amino‐1,3‐propanediol were co‐dissolved in DCM with triethylamine as a catalyst, and the mixture was reacted under a nitrogen atmosphere for 12 h. Then, the mixture was washed with distilled water and saturated salt solution for three times. The organic solvent was treated with excess magnesium sulfate and concentrated. Finally, the crude product was purified by silica gel column chromatography to get CNOH.

### Synthesis of PCP

PCP was synthesized as Figure S2A (Supporting Information). Briefly, CROH, COOH‐PEG_2K_‐COOH, EDC, and DMAP were co‐dissolved in anhydrous DMSO and allowed to react under a nitrogen atmosphere for 48 h. Then, the mixture was transferred into dialysis bags (MWCO: 5.0 kDa) and dialyzed against distilled water for 48 h to remove unreacted substances and catalysts. The product was finally lyophilized, and PCP was obtained and characterized by ^1^H NMR. PNP was synthesized as Figure S4A (Supporting Information) with the similar procedure, except CROH was replaced by CNOH.

### Preparation and Characterization of ECPCP

ES and PCP were co‐dissolved in DMSO, followed by copper chloride (CuCl_2_). The mixture was stirred for 2 h, and the unreacted CuCl_2_ was removed via centrifugation. The supernatant was dispersed in distilled water with continuous magnetic stirring for 2 h. Subsequently, the mixture was transferred into a dialysis bag (MWCO: 5.0 kDa) and dialyzed overnight. After concentration, ECPCP was obtained and stored at 4 °C. ECPNP was synthesized by the same procedure as ES and PNP, while EC was synthesized in the absence of PCP or PCP.

The particle size and zeta potential of the nanoparticles were measured by dynamic lighting scattering (DLS) with Malvern Zetasizer (Nano ZS, UK). The morphology of the nanoparticles was observed by transmission electrostatic microscopy (TEM, HT7700, Hitachi, Japan). ES content in ECPCP or ECPNP was measured using HPLC. The stability of the nanoparticles was evaluated by particle size. Briefly, the nanoparticles were dispersed in PBS (pH 7.4) or DMEM and stored at 37 °C. At pre‐determined time points, the particle size of the nanoparticles was measured by DLS.

### In Vitro Drug Release

Dialysis method was used to investigate the drug‐release behavior of the nanoparticles. Briefly, the nanoparticles with the same ES content were transferred into dialysis bags and placed in a shaking incubator with different conditions (100 mL, PBS pH 7.4, PBS pH 7.4 + 10 mm GSH, PBS pH 7.4 + 1% H_2_O_2_) at 37 °C. At pre‐determined time points, 1 mL of the samples was taken, and an equal volume of fresh medium was added. The drug content was measured by HPLC, and the accumulative drug release was calculated.

### In Vitro Cell Cytotoxicity Assay

MTT assay was used to investigate the cell cytotoxicity. Briefly, 4T1 cell was seeded into 96‐wells plates. After attachment, the culture medium was replaced by the medium containing nanoparticles with a gradient concentration of ES. After 24 h incubation, the cell cytotoxicity was measured by MTT. The absorbance was detected by Bio‐Rad plate reader at 570 nm, and the cell viability was calculated. The cell cytotoxicity of the nanoparticles in the presence of CuCl_2_ was also detected. Briefly, CuCl_2_ (1 µm or 100 nm) was added into the replacement‐medium, respectively.

### In Vitro Cell Apoptosis Assay

Annexin V‐FITC/PI apoptosis kit was utilized to investigate the apoptosis inducing effect of the nanoparticles. Briefly, 4T1 cells were seeded into 6‐wells plate and allowed attachment. Then, the nanoparticles were added (ES concentration: 20 µg mL^−1^) and incubated for 24 h. The cells were washed with PBS and collected into EP tube. Subsequently, Annexin V‐FITC and PI were added according to the protocols of the kit and eventually measured by CytoFLEX Flow Cytometry (Beckman Coulter, USA).

### In Vitro Cellular Uptake

Confocal laser scanning microscopy (CLSM) and flow cytometry were both used to investigate the cellular uptake of the nanoparticles. For CLSM assay, 4T1 cells were seeded into 6‐wells plate with cell climbing slices. After attachment, the culture medium was replaced by the serum‐free medium with couramin‐6 (C6) labeled nanoparticles. After 4 h incubation, the cells were washed with PBS and fixed with 4% polyformaldehyde (PFA). Then, the cells were stained with DAPI for visualizing the nucleus and observed by CLSM. For the flow cytometry assay, after incubation with the nanoparticles, the cells were washed with PBS and harvested by trypsin, which was finally measured by the flow cytometer.

### In Vitro Intracellular Cu Uptake

Intracellular Cu content was measured by copper (Cu) assay kits. Briefly, cells were seeded into 6‐wells plate to allow attachment. The nanoparticles were added and incubated for 6 h. Then, the cells were washed with PBS and harvested by trypsin. The intracellular Cu contents were measured according to the kit.

### In Vitro ROS and GSH Level

In vitro ROS and GSH levels were measured by ROS fluorometric assay kit and reduced glutathione (GSH) assay kit, respectively. Briefly, cells were seeded into 6‐wells plate to allow attachment, and the nanoparticles were added. After 6 h incubation, the culture medium was removed, and the cells were washed with PBS. In vitro ROS and GSH levels were detected according to the kits, respectively.

### In Vitro Cuproptosis Assay

Western blotting method was used to investigate the cuproptosis‐related protein expression. Briefly, 4T1 cells were seeded into 6 wells plate and treated with the nanoparticles for 24 h. RIPA cell lysate was used to extract the protein of cells, and the protein concentration was measured by BCA protein assay kit. Then, the loading buffer was added and heated for protein denaturation. The samples were added to sodium dodecyl sulfate‐polyacrylamide gel electrophoresis (SDS‐PAGE), and the proteins were transferred into the PVDF membrane. The PVDF membranes were blocked and successively incubated with primary antibodies (FDX1 and DLAT) and HRP‐labeled secondary antibodies. The images were obtained by Gel imaging system after culturing with ECL chemiluminescent reagent.

### In Vitro LPO and ROS Detection by CLSM

LPO was observed by the C11BODIPY fluorescent probe. Briefly, cells were seeded into glass‐covered 6 wells plates to allow attachment. The nanoparticles were added and incubated for 6 h. Then, cells were washed and treated with the C11BODIPY fluorescent probe. The cells were washed, fixed, and stained with DAPI. Finally, the fluorescence was observed by CLSM. DCFH‐DA was used as a fluorescent probe to detect intracellular ROS levels. The procedures were similar to LPO detection.

### Live/Dead Assay

Live/dead assay kit was used to investigate the cytotoxicity of different nanoparticles. Briefly, 4T1 cells were seeded into 6‐wells plate with cell climbing slices. After attachment, the nanoparticles were added and incubated for 24 h. Then, the cells were washed with PBS and incubated with Calcein AM/PI. The fluorescent was observed by CLSM.

### In Vitro ECPCP‐Induced ICD

For CRT expression assay, cells were seeded into 6‐wells plates. After attachment, the nanoparticles were added and incubated for 24 h. The cells were washed and fixed with 4% PFA. Then, the cells were labeled with anti‐CRT primary antibody and Alexa488‐conjugated secondary antibody. The fluorescence was observed by CLSM. Extracellular HMGB1 and ATP secretion were both measured with ELISA kits according to the manufacturer. For DCs maturation assay, 4T1 cells were first incubated with the nanoparticles for 6 h and co‐cultured with BMDCs for 24 h. Then, the cells were washed with PBS and harvested by trypsin. Matured DCs were analyzed by a cocktail antibody including anti‐CD11c‐FITC, anti‐CD86‐APC, and anti‐CD80‐PE.

### In Vivo Biodistribution Assay of ECPCP

Tumor‐bearing Balb/c mice were randomized in groups including EC, ECPNP, and ECPCP groups. The DiR‐labeled nanoparticles were intravenously administrated. At the pre‐determined time point, the mice were anesthetized and observed by IVIS imaging system. In the end, the mice were sacrificed and the major organs were harvested for imaging. The fluorescence intensity of the organs was calculated.

### In Vivo Antitumor Activity of ECPCP

Tumor‐bearing Balb/c mice were randomized in groups (*n* = 16) and treated with PBS, ES, EC, ECPNP, and ECPCP every other day for four times via tail vein (ES concentration: 5 mg kg^−1^). The tumor volume and the weight of the mice were monitored. Tumor volume was calculated. On the 14th day, six mice of each group were collected and sacrificed. The tumors were harvested and weighed. The major organs including heart, liver, spleen, lung, and kidney were obtained for H&E staining. The blood samples were collected for serum biochemical analysis. Other ten mice from each group were used to calculate the survival rate. For tumor tissues, the samples were fixed with formalin to prepare paraffin‐embedded slides for H&E staining, Ki67 immunohistochemistry staining and FDX1 immunofluorescence staining, respectively. The expression of FDX1 was also measured by western blot.

### In Vivo Cuproptosis and ICD Evaluation

*CRT Exposure*: For CRT immunofluorescence assay, tumor tissues were obtained to prepare frozen slides. Then, the slides were stained with CRT primary antibody and Alexa488‐conjugated secondary antibody. Finally, the slides were counter‐stained with DAPI to visualize the nucleus and observed by CLSM.

*Immune Cells and Cytokines Analysis*: Immune cell proportion was measured by a cocktail antibody method. The tumor tissues were obtained and digested into a single‐cell suspension. The cell suspension was added into the flow cytometry staining buffer (FCSB).

CD4^+^/CD8^+^ T cells and CD8^+^T secreted IFN‐γ were stained with CD3‐PerCP‐Cy5.5, CD4‐FITC, CD8‐PE, and IFN‐γ‐APC. CD4^+^ T cells, CD8^+^ T cells and IFN‐γ were gated as CD3^+^CD4^+^CD8^−^, CD3^+^CD4^−^CD8^+^ and CD3^+^CD4^−^CD8^+^IFN‐γ^+^, respectively. TAM population in tumor tissues was stained with CD11b‐FITC, CD206‐PE, and F4/80‐APC. M2 and M1 macrophages were gated as CD11b^+^F4/80^+^CD206^+^ and CD11b^+^F4/80^+^CD206^−^, respectively. For the matured DCs population in tumor‐draining lymph nodes (TDLNs), TDLNs were prepared as single‐cell suspension and stained with CD11c‐FITC, CD80‐PE, and CD86‐APC. Matured DCs were gated as CD11c^+^CD80^+^CD86^+^. For cytokines measurement, the tumor tissues were homogenized, and the supernatant was collected to measure IFN‐γ level by IFN‐γ ELISA kit.

*GSH and ROS Level Studies*: GSH and ROS levels of the tumor tissues were measured by ROS fluorometric assay kit and reduced glutathione (GSH) assay kit, respectively, as described in 2.12.

### Bilateral Antitumor Activity of ECPCP

To evaluate the antitumor immune response, a bilateral tumor‐bearing mice model was established as described in Figure 8D. Briefly, at day 7th, 4T1 cells were administrated into the left back of the mice. 4 days later, an equal amount of the 4T1 cells were administrated into the right back. Then, on days 0, 2, 4, and 6, the nanoparticles were intratumorally administrated into the primary tumor. The volume of both primary and distant tumors was measured every other day, and the mice were sacrificed on 14th day. The inhibition rate of both primary and distant tumors was calculated. Finally, the distant tumors were obtained for H&E staining and CD8^+^ T cells immunofluorescence staining, respectively.

### Statistical Analysis

Data were presented as mean ± SD. GraphPad Prism 8.0 software was used for statistical analysis. One‐way ANOVA was used to analyze the significant differences. “*”, “**” and “***” represented *p* < 0.05, *p* < 0.01 and *p* < 0.001, respectively.

## Conflict of Interest

The authors declare no conflict of interest.

## Author Contributions

H.W. and Z.Z. contributed equally to this work and are co‐first authors. H.W. and Z.Z. were responsible for all experiments and the original draft preparation. Y.C., L.Z., X.C., Y.H., Y.L., and H.W. were partly investigated in experiments and responsible for statistical data analysis. Y.H. and Y.L. were responsible for providing project‐related clinical information. Y.Z., H.L., and X.J. were responsible for the experiment design, conceptualization, writing‐review, validation, supervision, and financial support.

## Supporting information

Supporting Information

## Data Availability

The data that support the findings of this study are available from the corresponding author upon reasonable request.
