# Supplementary material for: A Self‐Amplifying ROS‐Responsive Nanoplatform for Simultaneous Cuproptosis and Cancer Immunotherapy
Source: Adv Sci (Weinh). 2024 Apr 3;11(23):2401047. doi: 10.1002/advs.202401047 (PMC11187900; doi:10.1002/advs.202401047)
Supplement: Supplementary file 1 — Supporting Information [file ADVS-11-2401047-s001.pdf]

ADVANCED SCIENCE
Open Access

# Supporting Information

for *Adv. Sci.*, DOI 10.1002/advs.202401047

A Self-Amplifying ROS-Responsive Nanoplatform for Simultaneous Cuproptosis and Cancer Immunotherapy

*Hangyi Wu, Zhenhai Zhang, Yanni Cao, Yuhan Hu, Yi Li, Lanyi Zhang, Xinyi Cao, Haitong Wen, Youwen Zhang*, Huixia Lv* and Xin Jin**

*Supporting Information for*

# A Self-Amplifying ROS-Responsive Nanoplatform for Simultaneous Cuproptosis and Cancer Immunotherapy

***Hangyi Wu[a,1], Zhenhai Zhang[b,1], Yanni Cao[a], Yuhan Hu[a], Yi Li[c], Lanyi Zhang[a], Xinyi Cao[a], Haitong Wen[a], Youwen Zhang[c,]*, Huixia Lv[a,]*, Xin Jin[c,]*.***

[a]Department of Pharmaceutics, China Pharmaceutical University, Nanjing 211198, China

[b]Jiangsu Province Academy of Traditional Chinese Medicine, Nanjing 210023, China

[c]Department of Pharmaceutics, The affiliated Suqian First People's Hospital of Nanjing Medical University, Suqian 223800, Jiangsu, China.

*Corresponding authors. Tel./fax: +86 15161291016 (Youwen Zhang); +86 13912965842 (Huixia Lv); +86 15951896676 (Xin Jin).

E-mail addresses: zyw_1016@163.com (Youwen Zhang); lvhuixia@163.com (Huixia Lv); jinxin871211@163.com (Xin Jin).

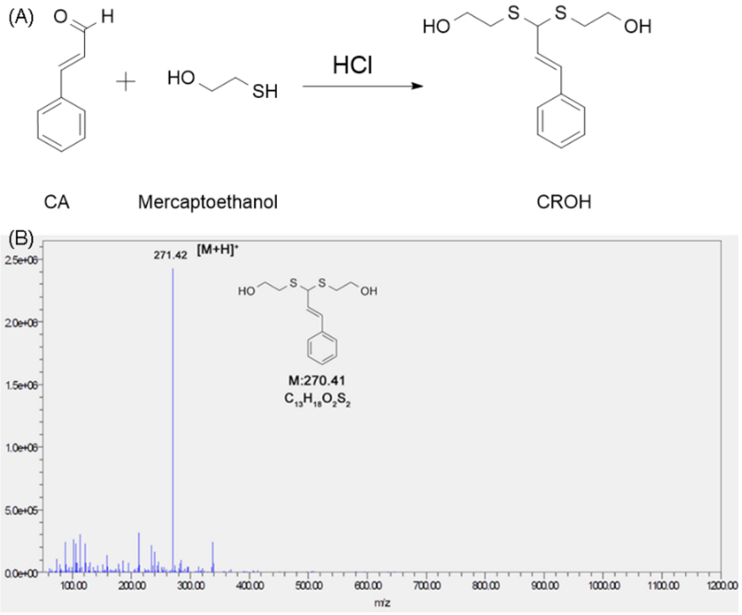


**Figure S1** (A) Synthesis pathway of CROH. (B) ESI-MS spectrum of CROH. $[M+H]^+ = 271.42$.

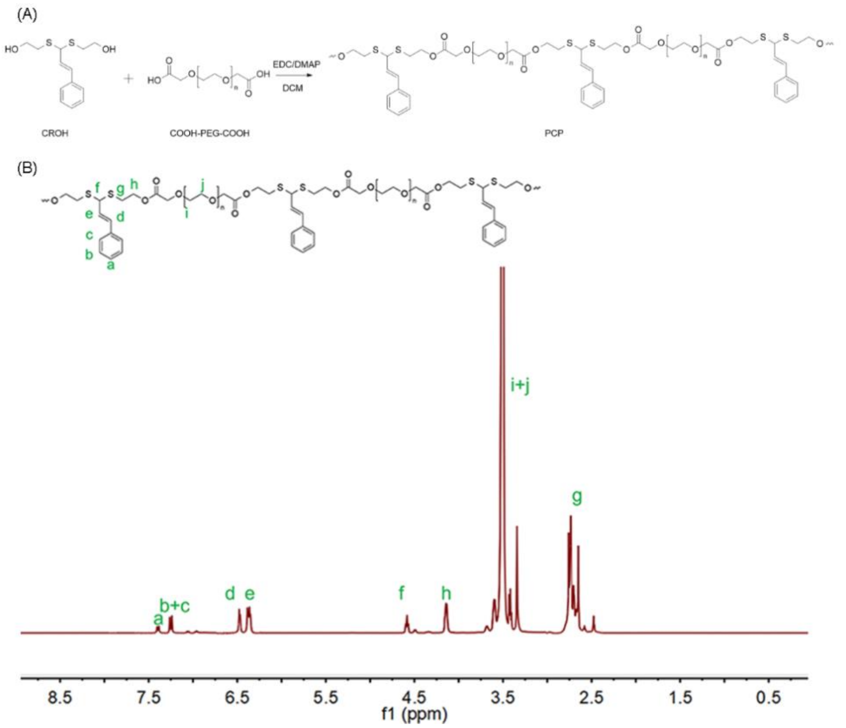


**Figure S2** (A) Synthesis pathway of PCP. (B) $^1$H NMR spectrum of PCP.

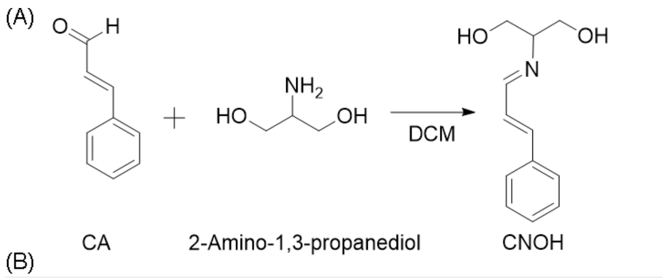


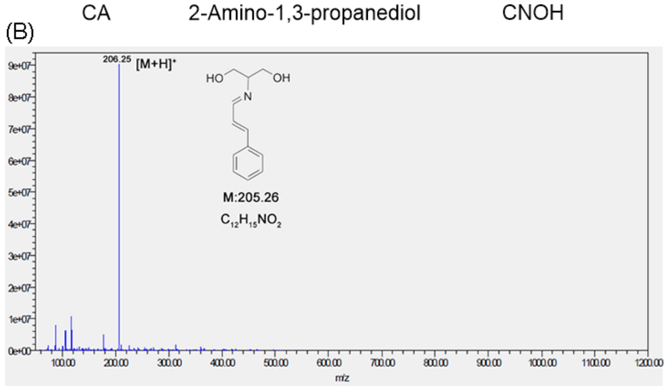


**Figure S3** (A) Synthesis pathway of CNOH. (B) ESI-MS spectrum of CNOH. $[M+H]^+ = 206.25$.

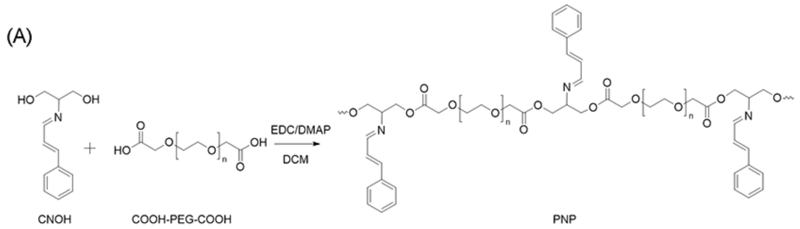


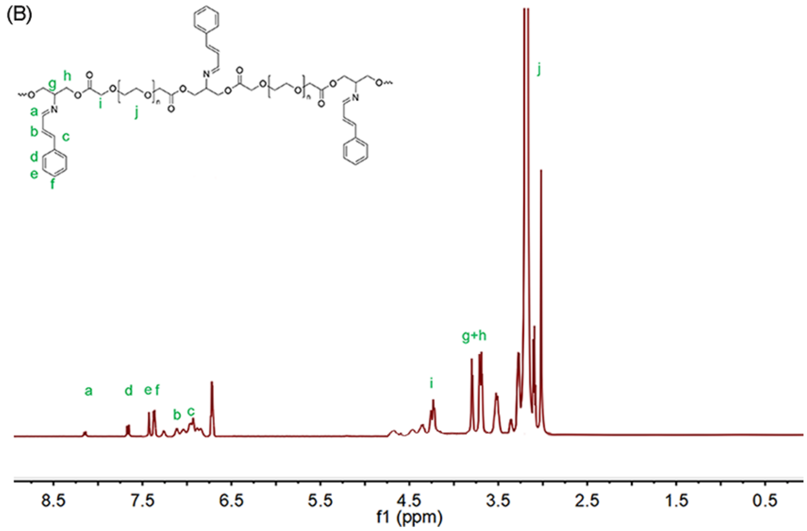


**Figure S4** (A) Synthesis pathway of PNP. (B) $^1$H NMR spectrum of PNP.

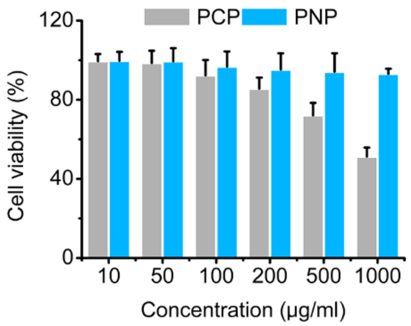


**Figure S5** *In vitro* cell cytotoxicity of PCP and PNP against 4T1 tumor cell line. Data are presented as mean ± SD, $n$ = 6.

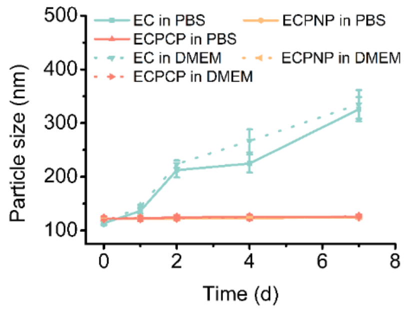


**Figure S6** *In vitro* stability of EC, ECPNP and ECPCP in PBS and DMEM at 37 °C. Data are presented as mean ± SD, $n$ = 3.

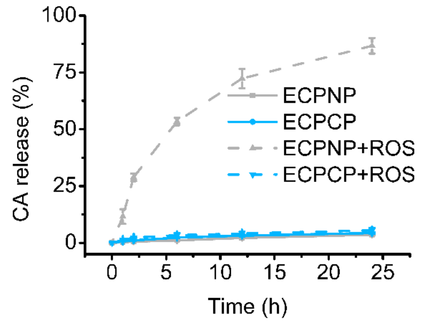


**Figure S7** *In vitro* CA release behavior from ECPNP and ECPCP under ROS condition at 37 °C. Data are presented as mean ± SD, $n$ = 3.

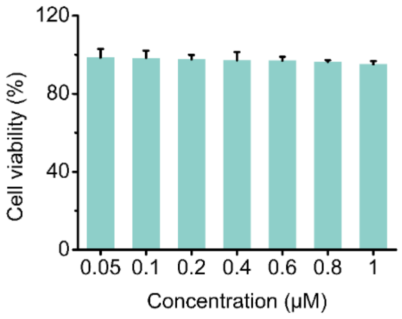


**Figure S8** *In vitro* cell cytotoxicity of $CuCl_2$ against 4T1 tumor cell line. Data are presented as mean ± SD, $n$ = 6.

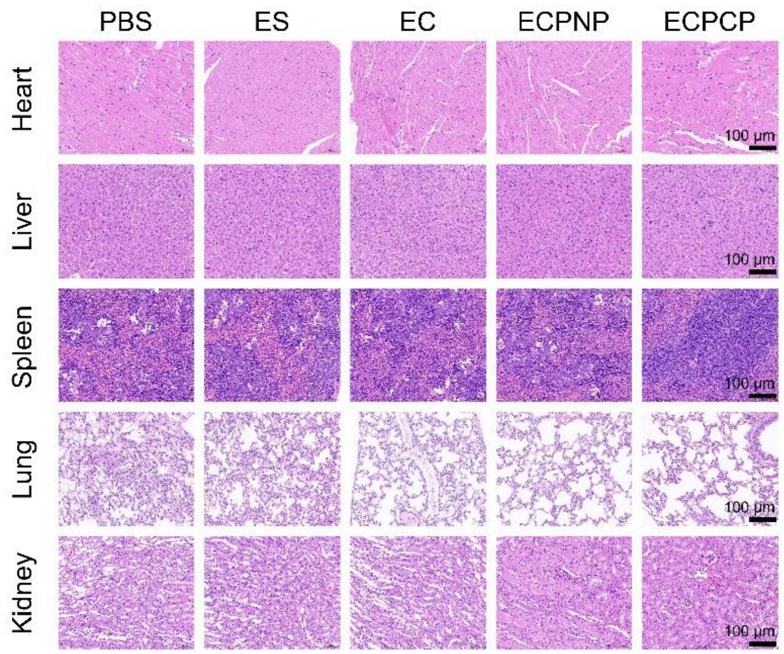


**Figure S9** H&E staining of major organs harvested from Balb/c mice after treatment. Scale bar: 100 μm.

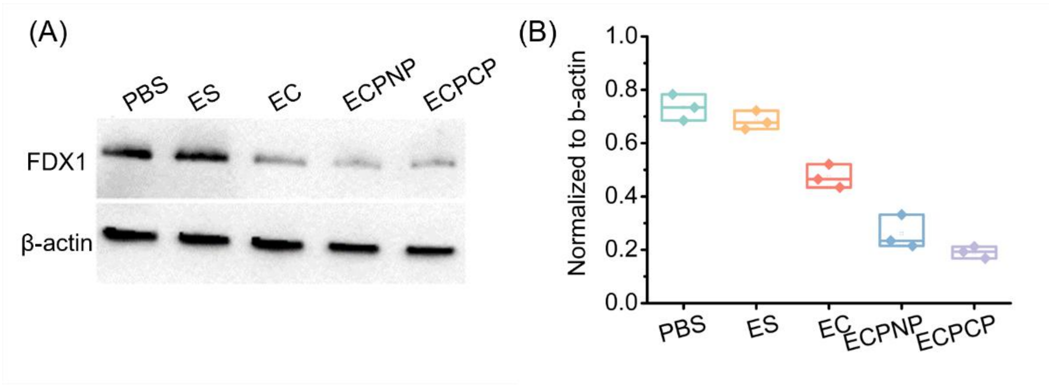


**Figure S10** The expression of FDX1 *in vivo* after treatments measured by western blot.

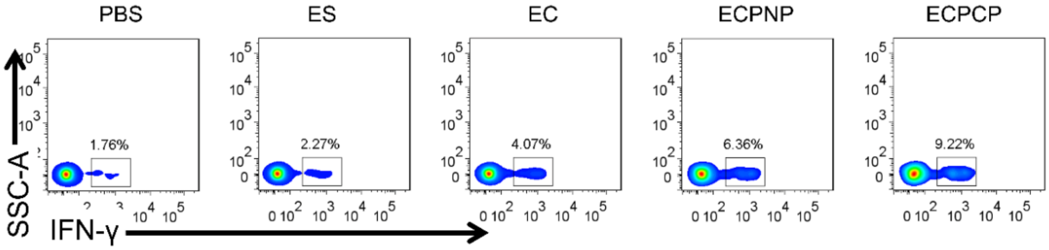


**Figure S11** Flow cytometry analysis of IFN-γ secretion by $CD3^{+}CD4^{-}CD8^{+}$ T lymphocytes.

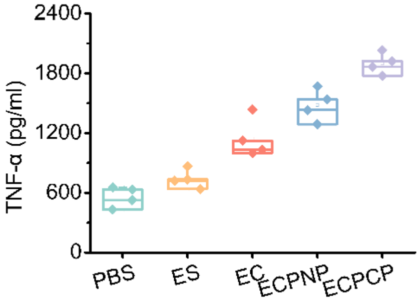


**Figure S12** *In vivo* TNF-α level in tumor tissues, respectively, $n = 4$.
